# Kinesins in Mammalian Spermatogenesis and Germ Cell Transport

**DOI:** 10.3389/fcell.2022.837542

**Published:** 2022-04-25

**Authors:** Mingxia Yao, Haoyang Qu, Yating Han, C. Yan Cheng, Xiang Xiao

**Affiliations:** ^1^ Center for Reproductive Health, School of Pharmaceutical Sciences, Hangzhou Medical College (Zhejiang Academy of Medical Sciences), Hangzhou, China; ^2^ Department of Urology and Andrology, Sir Run-Run Shaw Hospital, Zhejiang University School of Medicine, Hangzhou, China; ^3^ Zhejiang Provincial Laboratory of Experimental Animal’s & Nonclinical Laboratory Studies, Hangzhou Medical College, Hangzhou, China

**Keywords:** kinesin, testis, sertoli cells, spermatogenesis, cell junctions, blood-testis barrier, apical ectoplasmic specialization, cytoskeleton

## Abstract

In mammalian testes, the apical cytoplasm of each Sertoli cell holds up to several dozens of germ cells, especially spermatids that are transported up and down the seminiferous epithelium. The blood-testis barrier (BTB) established by neighboring Sertoli cells in the basal compartment restructures on a regular basis to allow preleptotene/leptotene spermatocytes to pass through. The timely transfer of germ cells and other cellular organelles such as residual bodies, phagosomes, and lysosomes across the epithelium to facilitate spermatogenesis is important and requires the microtubule-based cytoskeleton in Sertoli cells. Kinesins, a superfamily of the microtubule-dependent motor proteins, are abundantly and preferentially expressed in the testis, but their functions are poorly understood. This review summarizes recent findings on kinesins in mammalian spermatogenesis, highlighting their potential role in germ cell traversing through the BTB and the remodeling of Sertoli cell-spermatid junctions to advance spermatid transport. The possibility of kinesins acting as a mediator and/or synchronizer for cell cycle progression, germ cell transit, and junctional rearrangement and turnover is also discussed. We mostly cover findings in rodents, but we also make special remarks regarding humans. We anticipate that this information will provide a framework for future research in the field.

## Introduction

Kinesins, dyneins, and myosins are three large superfamilies of motor proteins that are involved in intracellular transport. Kinesins and dyneins, which are powered by the hydrolysis of adenosine triphosphate (ATP), generally move in opposite directions along microtubule tracks, toward the microtubule plus and minus ends (i.e., away from or towards the center of the cell), respectively. Myosins, on the other hand, hydrolyze ATP to enable its movement on filamentous actin (F-actin), towards the fast-growing “barbed” end (plus end) of the polar microfilaments [for reviews (Kato et al., 2018; Sweeney and Holzbaur, 2018),]. Here, we focus on kinesin superfamily proteins (KIFs) and their evolving functions in mammalian spermatogenesis.

**FIGURE 1:**
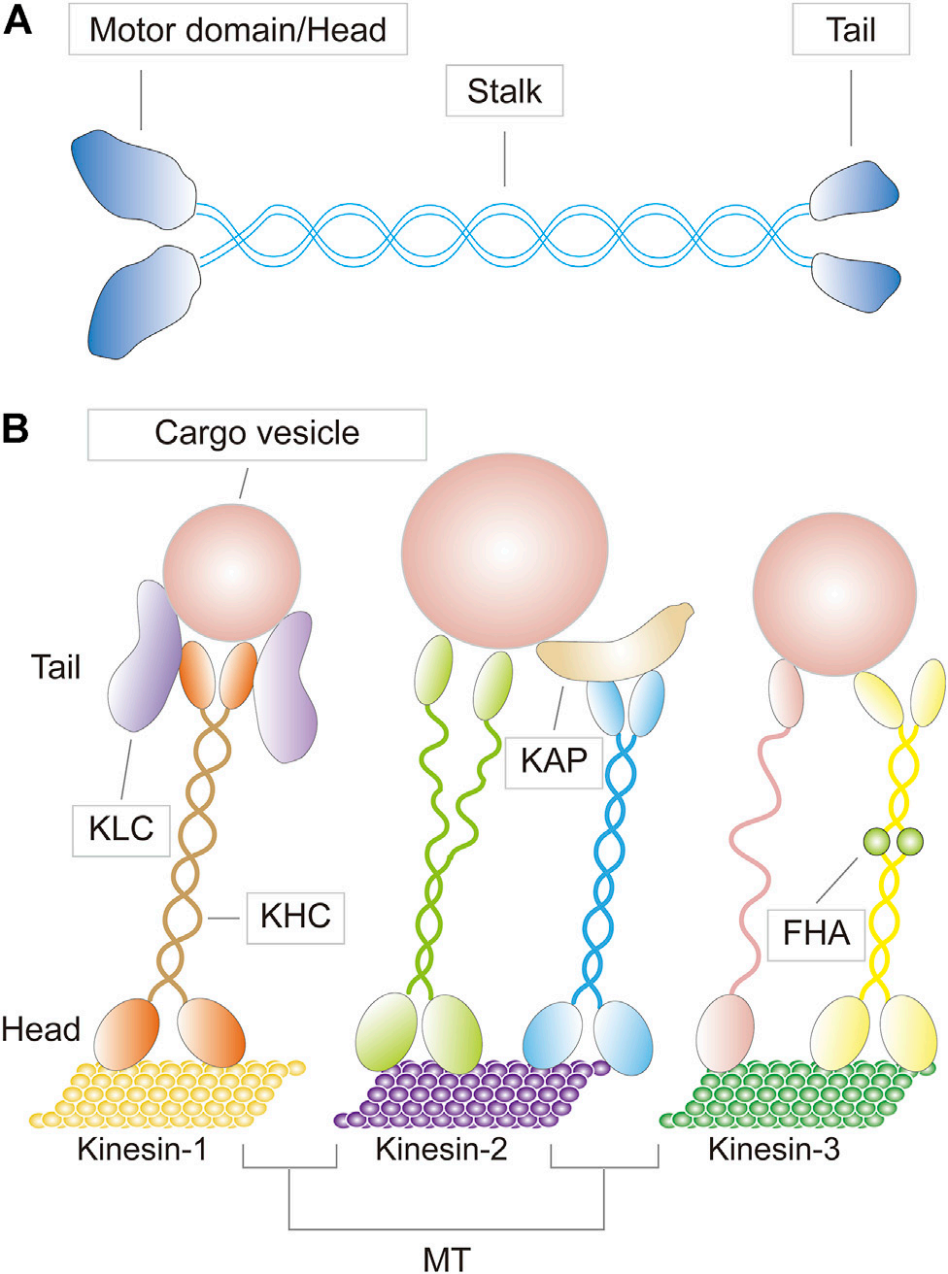
**(A)** A schematic diagram depicting the basic structure of a kinesin motor **(B)** Three representative kinesins (kinesin-1,-2, and -3) bearing cargoes traveling on microtubule (MT) tracks, with their specific molecular structures shown. KLC, kinesin light chain; KHC, kinesin heavy chain; KAP, kinesin associated protein; FHA, forkhead associated domain.

**FIGURE 2:**
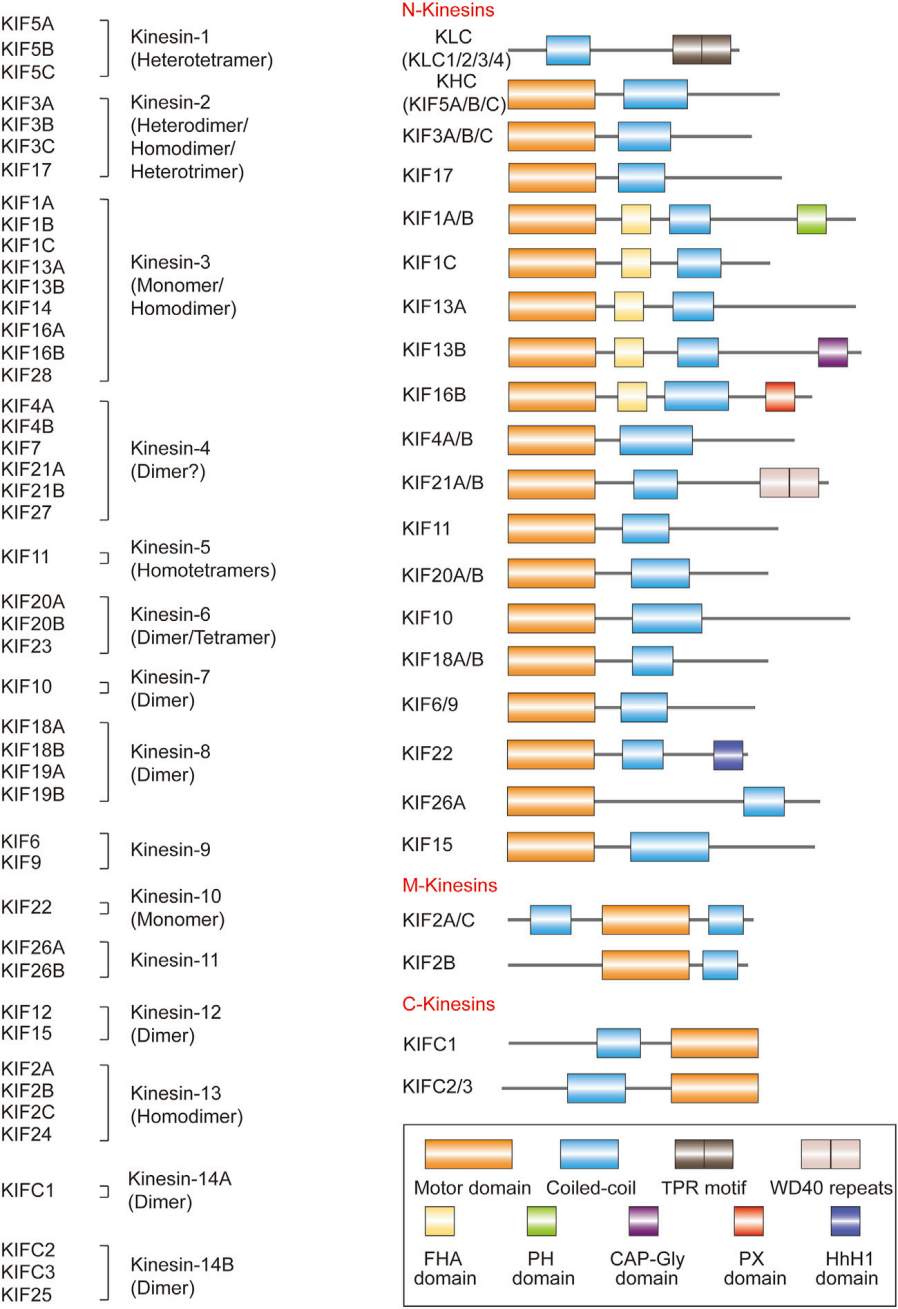
The domain structure and classifications of kinesins discovered in each of the human, mouse, and rat genomes (except that KIF16A and KIF19B are predictive in the rat). KIF11 is also known as KIF8 in the mouse, and KIF6 is also known as kinesin-related protein 3 (KRP3) in the rat. KIFC1 has isoforms KIFC4 and KIFC5 in the mouse. Kinesins share a common structure but also have domains that are unique to each KIF member. KLC, kinesin light chain; KHC, kinesin heavy chain; TPR, tetratricopeptide repeat; WD40 repeats, protein interaction motifs of approximately 40 amino acids that usually terminate in tryptophan-aspartic acid (WD); FHA, forkhead associated; PH, Pleckstrin homology; CAP-Gly, cytoskeleton-associated protein glycine-rich; PX, PhoX homologous; HhH1, Helix-hairpin-Helix DNA-binding motif class 1.

The first kinesin (also known as conventional kinesin or kinesin-1) was discovered in 1985 (Vale et al., 1985; Brady, 1985). Since then, dozens more have been identified, and before a unified nomenclature was proposed in 2004, there were numerous designations and redundant names for a single KIF (Lawrence et al., 2004; Endow et al., 2010). For example, KIF23, a member of the kinesin-6 family, was also known as CHO1/MKLP1 (human), ZEN-4/CeMKLP1 (*C. elegans*), and Pavarotti/MKLP1 (Pav-KLP, *Drosophila*) (Kuriyama et al., 2002). KIFs appear to be present in all eukaryotes, from fungi to plants and animals, as well as in different cell types. A typical KIF molecule has a motor domain—the head—that contains the ATP and microtubule binding sites and is referred to as the catalytic core; a tail domain opposite the motor that is involved in adaptor or cargo binding; and a long filamentous coiled-coil stalk in between the head and the tail that allows oligomerization and self-interaction, as well as regulation of microtubule binding and subcellular localization of the motor and cargo (Figure 1) (Miki et al., 2001; Marx et al., 2009; Hirokawa and Tanaka, 2015; Hirokawa et al., 2020). The motor domains of KIFs share considerable homology, which defines the superfamily. Outside of this conserved region, KIF members have minimal commonalities, with each carrying class-specific domains that recognize different payloads. KIFs come in a variety of shapes, including monomers, heterologous or homologous dimers, trimers, and tetramers (Figure 2). For instance, the founding member of the superfamily, kinesin-1, is a heterotetrameric motor made up of two kinesin heavy chain (KHC) and two kinesin light chain (KLC) subunits. The motor domain is housed in the KHCs, which interacts with the KLCs that connects to the cargo (Figure 1). KIFs have been shown to transport a wide variety of cargoes through the cell, including proteins, lipid droplets, mRNAs, chromosomes, organelles, and vesicles such as mitochondria, endoplasmic reticulum, early endosomes, late endosomes, and lysosomes. They have a role in pathogenesis of metabolic diseases, brain development, neurotransmitter transport, cilia and flagella assembly, and chromosome and spindle function during mitosis and meiosis. To date, over 45 mouse and human KIFs have been identified, which are further divided into 14 classes of three major types based on the position of the motor domain, namely N-kinesins with the motor domain in the N-terminal region (kinesin-1–12), M-kinesins with the motor domain located internally in the middle of the polypeptide chain (kinesin-13), and C-kinesins with the motor domain located C-terminally (kinesin-14) (Figure 2). Interestingly, the direction of cargo transport mirrors this functional classification, with N- and C-kinesins driving microtubule plus and minus end-directed transport, respectively, whereas M-kinesins displaying non-motor activities of depolymerizing microtubules (Miki et al., 2001; Marx et al., 2009; Hirokawa and Tanaka, 2015; Hirokawa et al., 2020). Some N- and C-kinesins also perform non-motor functions. Kinesin-8, for example, is a depolymerizing kinesin that regulates the length and disassembly of microtubules at the plus-ends (Ohi et al., 2013). Kinesin-5 has crosslinking and sliding properties for anti-parallel microtubules (Kapitein et al., 2005; van den Wildenberg et al., 2008).

Using Northern blotting, PCR analysis with cDNA, and/or immunoblotting, most subfamilies of KIFs have been shown to be preferentially expressed in rodent testes (Table 1). Many KIFs, including KIF6, KIF7, KIF9, KIF17B, KIF18B, KIFC1, and KIFC3, are predominantly, if not exclusively, expressed in the testis (Nakagawa et al., 1997; Miki et al., 2001; Noda et al., 2001; Yang and Sperry, 2003; Wong-Riley and Besharse, 2012). The presence of such large amounts of KIFs in the testis clearly indicates that the testis has a very robust intracellular transport system that relies on KIF participation. Mammalian spermatogenesis, which occurs in the testis, is one of the most important developmental processes in nature. During this process, KIFs are involved in spindle formation and chromosome segregation during meiosis, acrosome biogenesis, nuclear shaping of the sperm head, flagellar movements, spermatid transcription, and spermatid transport within Sertoli cell crypts (Vogl et al., 2008; Tang et al., 2016a; Ma et al., 2017). Mutations in KIFs have been shown to impact male fertility in mouse models (Table 2). Their function in the mammalian testis, however, is unclear. Although the testis contains a large number of mitotically and meiotically active germ cells, as well as highly polarized Sertoli cells as one type of specialized epithelial cell, KIF has received less attention in testicular biology than in mitosis or other polarized cell settings such as neurons and epithelial/endothelial cells. The goal of this review is to summarize recent KIF findings related to mammalian spermatogenesis. We discuss herein the significance of this superfamily of microtubule motor proteins in the testis, with a focus on their probable function in the remodeling of the blood-testis barrier (BTB) and the Sertoli cell-spermatid junction dynamics to facilitate germ cell transport. We anticipate that this information will serve as a basis for future studies in the field.

**TABLE 1:** KIFs discovered in rodent testes.

KIF/subunit		Testicular localization	Detection technique(s)	Reference(s)
Kinesin-1	KIF5A	Somatic testicular cells (expressed before meiosis)	RT-PCR	Junco et al. (2001)
	KIF5B	Spermatocytes, Sertoli cells	RT-PCR, WB, IF, scRNA-seq	(Junco et al., 2001; Jung et al., 2019)
	KIF5C	Spermatocytes, round spermatids (extremely low level), acrosome of spermatozoa	RT-PCR, WB, IF	(Junco et al., 2001; Mannowetz et al., 2010)
	KLC1/2	Pachytene spermatocytes	RT-PCR	Junco et al. (2001)
	KLC3	Spermatocytes, spermatids (highest in round spermatids), spermatozoa, sperm tails	RT-PCR, WB, IF	
Kinesin-2	KIF3A/B (KIF3/KAP3 complex/Kinesin-II)	Cytoplasm in pachytene spermatocytes, round spermatids and elongating spermatids; basal body and axoneme of round and elongating spermatids, manchette of elongating spermatids, sperm tails; Sertoli cell trans-Golgi network	RT-PCR, WB, IF	(Kondo et al., 1994; Yamazaki et al., 1995; Huang et al., 2012; Lehti et al., 2013)
	KIF17B	Nucleus and cytoplasm of round spermatids, elongating spermatids, manchette, chromatoid bodies of round spermatids, principal piece of the sperm tail	NB, WB, IF	(Macho et al., 2002; Chennathukuzhi et al., 2003; Kotaja et al., 2006; Saade et al., 2007)
Kinesin-3	KIF13A/B KIF16A/B	N/A	NB	(Nakagawa et al., 1997; Miki et al., 2001)
Kinesin-4	KIF7	N/A	NB	Nakagawa et al. (1997)
	KIF27	Cytoplasm of all germ cells, perinuclear ring of the manchette, HTCA, residual bodies	ISH, IF (KIF27GFP-transgenic mouse)	Nozawa et al. (2014)
Kinesin-5	KIF11	Spermatogonia and spermatocytes	WB, IF	(Birtel et al., 2017; Hara-Yokoyama et al., 2019)
Kinesin-6	KIF20A/B	Sertoli cells, germ cells, ectoplasmic specialization	WB, IF, microarrays, RT-PCR	(Vaid et al., 2007; Wu et al., 2021a)
	KIF23	Germ cell intercellular bridges	RT-ddPCR, WB, IF	(Greenbaum et al., 2007; Vikberg et al., 2020)
Kinesin-7	KIF10	Spermatocytes	IF, CM	(Kallio et al., 1998; Eaker et al., 2001)
Kinesin-8	KIF18A/B	Sertoli cells, germ cells	NB, RT-PCR	(Miki et al., 2001; Luboshits and Benayahu, 2005; Czechanski et al., 2015; Wu et al., 2021a)
	KIF19A	N/A	NB	Miki et al. (2001)
Kinesin-9	KIF6/KRP3	Spermatogonia and/or Sertoli cells, pachytene spermatocytes, round spermatids	NB, ISH, WB, IF	(Nakagawa et al., 1997; Zou et al., 2002)
	KIF9	Spermatocytes, spermatids, sperm flagellum, Sertoli cells	RT-PCR, WB, In silico expression analysis, IF	(Miyata et al., 2020; Wu et al., 2021a)
Kinesin-10	KIF22	N/A	NB	(Tokai et al., 1996; Yang et al., 1997)
Kinesin-12	KIF15	Sertoli cells, germ cells; apical and basal apical ectoplasmic specialization, BTB	NB, RT-PCR, WB, IF	(Nakagawa et al., 1997; Wu et al., 2021a)
Kinesin-13	KIF2B	N/A	NB	Miki et al. (2001)
	KIF24			
Kinesin-14A	KIFC1	MBOs in the medulla of early spermatids, acrosome of elongating spermatids, spermatid manchette, nucleus of early spermatids, distal cytoplasm/residual bodies, Sertoli cells	RT-PCR, WB, IF, EM	(Navolanic and Sperry, 2000; Yang and Sperry, 2003; Wu et al., 2021a)
	KIFC4A	N/A	NB	Yang et al. (1997)
	KIFC5A/B/C	Spermatid manchette and sperm flagella	RT-PCR, NB, IF	Navolanic and Sperry, (2000)
Kinesin-14B	KIFC3	N/A	NB, WB	Noda et al. (2001)

Abbreviations: RT-PCR, reverse transcription-polymerase chain reaction; WB, western blot; IF, immunofluorescence; scRNA-seq, single-cell RNA-sequencing; N/A, not available; NB, northern blot; HTCA, head-tail coupling apparatus; ISH, *in situ* hybridization; ddPCR, droplet digital PCR; CM, confocal microscopy; BTB, blood-testis barrier; MBOs, membrane-bounded organelles; EM, electron microscopy.

**TABLE 2:** Phenotypes in KIF mutant mice/embryos^a^.

KIF	Mouse mutant	Viability	Fertility	Phenotype	Reference(s)
Kinesin-1					
KIF5A	KIF5A^−/−^	Neonatal lethal		Unexpanded lungs;	Xia et al. (2003)
				No obvious pathological changes in the brain;	
				Nuclei and cell bodies of spinal cord motor neurons appeared to be bigger than motor neurons of control littermates	
	Postnatal KIF5A cKO in neurons	Postnatal lethality by 3-4 weeks of age		Normal in appearance;	(Xia et al., 2003; Nakajima et al., 2012)
				Spontaneous epileptic seizure;	
				Neurofilament (NF) transport defects;	
				Impairment of GABAAR trafficking in neurons	
**KIF5B**	KIF5B^−/−^	Embryonic lethal (died between 9.5–11.5 dpc)		Severe growth retardation;	Tanaka et al. (1998)
				Impaired lysosomal dispersion;	
				Abnormal Perinuclear Clustering of Mitochondria	
	Postnatal KIF5B cKO in neurons	Viable	Fertile	Normal in general appearance or brain size;	Zhao et al. (2020)
				Deficits in dendritic transport, synaptic plasticity and memory	
	**KIF5B KD in testis (shRNAs)**	Viable	**Infertile (male)**	Defects in cell-cell adhesion in the seminiferous epithelium;	Sehgal et al. (2014)
				Diminished levels of plakoglobin, PKP3 and DSC2 and DSC3 at the cell border	
KIF5C	KIF5C^−/−^	Viable	Fertile	Normal in appearance;	Kanai et al. (2000)
				Smaller brain size;	
				Relative loss of motor neurons to sensory neurons	
KLC1	KLC1^−/−^	Viable	Hemizygotes are fertile	Small size and brain; Overt movement defects;	(Rahman et al., 1999; Falzone et al., 2009; Saez et al., 2020)
				Obvious alterations in the intracellular localization of KHC in neurons;	
				A marked depletion of KLC2 in the sensory neuron cell bodies;	
				Abnormal Tau phosphorylation;	
				Axonopathies with cytoskeletal disorganization and abnormal cargo accumulation;	
				Defects in axonal transport of CB1R vesicles; impaired cofilin activation in cerebral cortex	
**KLC3**	**High level expression of transgenic KLC3 deletion mutant in spermatids**	Viable	**Subfertile (male)**	Reduced sperm count;	Zhang et al. (2012)
				Produce sperm with impaired motility;	
				Elongating spermatids exhibit midpiece abnormalities	
Kinesin-2					
**KIF3A**	KIF3A^−/−^	Embryonic lethal (died at 10 dpc)		Randomization of left-right asymmetry; Situs inversus; embryonic ciliary morphogenesis defects	Marszalek et al. (1999)
	**KIF3A cKO in male germ cells**	Viable	**Infertile (male)**	No changes in overall organization of the seminiferous epithelium;	Lehti et al. (2013)
				Defective morphology of the sperm head and tail;	
				Abnormally long manchette and delayed clearance of the manchette	
	KIF3A cKO in renal tubular epithelial cells	Viable;		Cysts begin to develop in the kidney at P5 and cause renal failure by P21;	Lin et al. (2003)
		By P35 exhibited lethargy and growth retardation		Cyst epithelial cells lacked primary cilia and exhibited increased proliferation and apoptosis, apical mislocalization of the EGFR, increased expression of β-catenin and c-Myc, and inhibition of p21^Cip1^	
	KIF3A cKO in airway epithelial cells	Viable;		Marked decrease in acetylated α-tubulin staining;	Giridhar et al. (2016)
				Impaired barrier function, epithelial repair, innate immune responses, and mucociliary clearance	
KIF3B	KIF3B^−/−^	Embryonic lethal at midgestation		Randomization of left-right asymmetry;	Nonaka et al. (1998)
				Growth retardation, pericardial sac ballooning, and neural tube disorganization;	
				Disrupted ciliogenesis	
KIF3C	KIF3C^−/−^	Viable	Fertile	Apparently developed normally	Yang et al. (2001a)
KIF17	germ-line KIF17^−/−^	Viable	Fertile	Healthy and indistinguishable from wild-type mice	Jiang et al. (2015)
Kinesin-3					
KIF1A	KIF1A^−/−^	Neonatal lethal		Smaller size;	Yonekawa et al. (1998)
				Motor and sensory disturbances;	
				Decreased synaptic vesicle precursor transport;	
				Marked neuronal degeneration and neuronal cell death	
KIF1B	KIF1B^−/−^	Died at birth		Smaller size;	Zhao et al. (2001)
				Displayed multiple neurological abnormalities	
KIF1C	KIF1C^−/−^	Viable	Fertile	No obvious abnormalities	(Nakajima et al., 2002; Nakajima and Tanaka, 2010)
KIF13A	KIF13A^−/−^	Viable	Fertile	Elevated anxiety-related behavioral phenotypes	(Zhou et al., 2013; Mills et al., 2019)
KIF13B	KIF13B^−/−^	Viable	Fertile	No gross anomalies and normal body size and weight;	Kanai et al. (2014)
				Increased levels of serum cholesterol and factor VIII;	
				Decreases LRP1-mediated endocytosis	
	Expression of a truncated form of KIF13B lacking the CAP-Gly domain at the C terminus (KIF13BΔCG)	Viable	Fertile	Significantly elevated levels of total cholesterol and LDL; relatively increased HDL; Elevated levels of plasma factor VIII activity;	Mills et al. (2019)
				Subcellular mislocalization of truncated KIF13B concomitant with the mislocalization of LRP1	
KIF14	KIF14^−/−^	Postnatal lethality (died before weaning)		Severe brain malformation and hypomyelination;	(Fujikura et al., 2013; Moawia et al., 2017; Reilly et al., 2019)
				Growth retardation and a reduced brain volume (microcephaly)	
Kinesin-4					
KIF7	KIF7^−/−^	Died at birth		Showed edema, exencephaly, and polydactyly	Cheung et al. (2009)
KIF21A	KIF21A^−/−^ (motor truncation)	Died within 24 h of birth		Harbored a very low level (<15% of the WT protein level) of a truncated Kif21a missing the motor domain necessary for motor-microtubule interaction and anterograde movement	Cheng et al. (2014)
KIF21B	KIF21B^−/−^	Viable	Fertile	No apparent gross abnormalities;	Morikawa et al. (2018)
				Impaired Fear Extinction	
Kinesin-5					
KIF11	KIF11^−/−^	Early embryonic lethal		Embryo was morphologically smaller and partially resorbed	(Castillo and Justice, 2007; Chauvière et al., 2008)
	KIF11 cKO in postnatal vascular endothelial cells	Viable	Fertile	Severely stunted growth of the retinal vasculature, mildly stunted growth of the cerebellar vasculature	Wang et al. (2020b)
Kinesin-6					
KIF20A	KIF20A germline cKO/inducible KO in NPCs	Embryonic lethal		Embryos showed smaller body and brain sizes as well as reduced cortical thickness; Thinner neuronal layer (βIII-tubulin) in the cortex;	Geng et al. (2018)
				Loss of cortical NPCs; Defect in neurogenesis	
KIF20B	A loss-of-function mutant with an undetectable level of KIF20B protein	Perinatal lethality		Fully penetrant microcephaly;	Janisch et al. (2013)
				Brains displayed defects in cytokinetic abscission;	
				Reduced neurogenesis but preserved lamination;	
				Increased apoptosis during early cerebral cortex development	
KIF23	KI model of the human KIF23 p.P916R mutation causing CDA III	Viable	Fertile	Healthy, active, lacked characteristic features of human CDA III and grew old without any signs of any disease	Vikberg et al. (2020)
Kinesin-7					
KIF10	KIF10^−/−^	Embryonic lethal (died between 7.5/8.5 dpc)		Developmental arrest;	Putkey et al. (2002)
				Massive chromosome segregation defects	
Kinesin-8					
**KIF18A**	**KIF18A** ^**−/−**^	Viable	**Infertile(male)**; fertile (female)	Reduced growth rates and increased prewean mortality;	(Liu et al., 2010; Zhu et al., 2013; Fonseca et al., 2019)
				Testis atrophy;	
				Severe developmental impairment of seminiferous tubules;	
				Perturbed microtubule dynamics and spindle pole integrity;	
				Chromosome congression defects during mitosis and meiosis;	
				Impaired Akt phosphorylation;	
				KO mice were protected from CAC;	
				Tumor cells from KO mice underwent more apoptosis	
	A missense R308K mutation in the motor domain of KIF18A (EMS induced *Kif18a* ^*gcd2*^ mutant)	Viable	Strain dependent sterility	Overtly normal with the exception of sterility;	Czechanski et al. (2015)
				Testes have few or no developing germ cells and ovaries are small with few or no follicles;	
				Chromosome alignment defects in somatic and germ cells;	
				A germ cell specific, checkpoint-mediated mitotic arrest	
KIF19A	KIF19A^−/−^	Viable	Infertile (female)	Growth retardation and higher mortality;	Niwa et al. (2012)
				Hydrocephalus; fallopian tube obstruction;	
				Abnormally elongated cilia that could not generate proper fluid flow	
Kinesin-9					
KIF6	C- and N-terminal truncating mutation in KIF6	Viable	Fertile	Severe progressive hydrocephalus, loss of ependymal cell cilia	Konjikusic et al. (2018)
**KIF9**	**KIF9 large deletion mutant**	Viable	**Subfertile (male)**	No overt abnormalities including hydrocephalus;	Miyata et al. (2020)
				Impaired sperm motility;	
				Partially impaired ZP penetration	
Kinesin-10					
KIF22	KIF22^−/−^	Viable	Fertile	Half of the embryos died prior to E9.5, but the surviving embryos developed into healthy, fertile adult mice;	Ohsugi et al. (2008)
				No histological abnormality was found in the testis, and the motility of the sperms of male mice was normal	
Kinesin-11					
KIF26A	KIF26A^−/−^	Postnatal lethality at approximately 2 weeks of age		Exhibited growth retardation;	(Zhou et al., 2009; Wang et al., 2018)
				Developed a megacolon with enteric nerve hyperplasia;	
				Prolonged and enhanced nociceptive responses;	
				Hyperbranched DRG axons and their prolonged Ca transients;	
				Hyperphosphorylation of FAK and PMCA;	
				SFK inhibitor PP2 reverses KO phenotypes *in vitro* and *in vivo*	
KIF26B	KIF26B^−/−^	Died within 24 h of birth		Kidney agenesis;	Uchiyama et al. (2010)
				Reduced expression of integrin α8 and N-cadherin in the adhesion of mesenchymal cells to ureteric buds in metanephros	
Kinesin-12					
KIF12	KIF12^−/−^	Viable	Fertile	Suffered from hypoinsulinemic glucose intolerance due to increased beta cell oxidative stress	Yang et al. (2014)
Kinesin-13					
KIF2A	KIF2A^−/−^	Died within one day of birth without sucking milk		Multiple brain abnormalities;	(Homma et al., 2003; Maor-Nof et al., 2013)
				Neuronal migratory defects;	
				Lower microtubule depolymerizing activity;	
				Severe skin hyperinnervation by sensory axons	
	Postnatal KIF2A cKO induced by tamoxifen injections beginning at postnatal week 3	Died by postnatal week 6		Weight loss, hyperactivity, and eventually death with an epileptic hippocampus;	Homma et al. (2018)
				DGCs showed dendro-axonal conversion, leading to the growth of many aberrant overextended dendrites that eventually developed axonal properties	
Kinesin-14A					
KIFC1	KIFC1^−/−^	Viable	Fertile	N/A	Hirokawa et al. (2010)
Kinesin-14B					
KIFC2	KIFC2^−/−^	Viable	Fertile	Apparently developed normally	Yang et al. (2001b)
KIFC3	KIFC3^−/−^	Viable	Fertile	Apparently developed normally	(Yang et al., 2001c; Xu et al., 2002)

a This table is not intended to be exhaustive. Although there is limited and preliminary information on KIF function during mammalian spermatogenesis, it illustrates the importance of KIFs in fertility and development. Fertility refers to both sexes unless otherwise stated. KIFs with potential effects on male fertility, as well as their corresponding mice mutants and fertility, are highlighted in bold. Readers can look up a mutation in the KIF protein by its name to see whether it affects fertility. Furthermore, based on what has been discovered in other cellular systems, it is an excellent source of inspiration for study on Sertoli-germ cell adhesion and germ cell transport, e.g., the involvement of KLC1 in Tau phosphorylation and cofilin activation. Refer to Tables in reference (Ma et al., 2017) for details on KIF roles during different stages of germ cell development (including non-mammals) and reference (Wu et al., 2021b) for a summary of pathological conditions caused by KIF mutations in humans. Abbreviations: cKO, conditional knockout; KD, knockdown; PKP3, plakophilin 3; DSC, desmocollin; P, postnatal day; EGFR, epidermal growth factor receptor; LDL, Low-density lipoprotein; LRP1, LDL-receptor Related Protein 1; HDL, High-density lipoprotein; NPCs, neural progenitor cells; NSC, neural stem cell; KI, knock-in; CDA III, congenital dyserythropoietic anemia type III; CAC, colitis-associated colorectal tumor; gcd2, germ cell depletion 2; EMS, ethylmethanosulfonate; ZP, zona pellucida; DRG, dorsal root ganglia; PMCA, plasma membrane Ca^2+^ ATPase; DGCs, dentate granule cells; N/A, not available.

## Kinesins in Germ Cell Development in Mammalian Testes

### Cell Division Regulation

**FIGURE 3:**
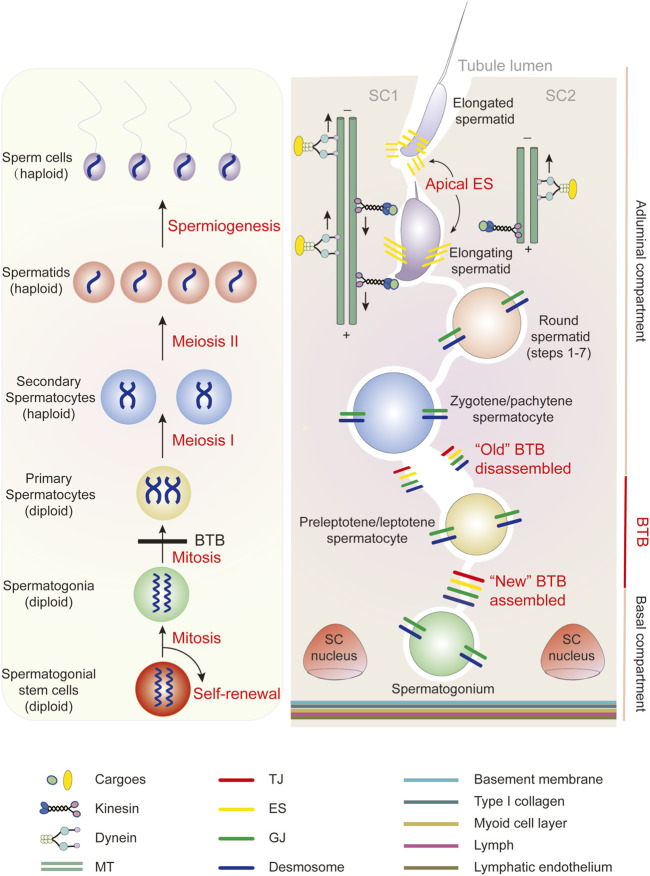
(Left) A diagram depicting the cell cycle progression and different germ cell types during spermatogenesis. (Right) A schematic drawing that illustrates the cross section of the seminiferous tubule in the rat testis. Four types of Sertoli cell-cell junctions at the blood-testis barrier (BTB), namely tight junction (TJ), basal ectoplasmic specialization, gap junction (GJ), and desmosome, as well as cell-cell junctions at the Sertoli-germ cell interface are illustrated. Spermatogonia, spermatocytes, and steps 1–7 round spermatids are connected to Sertoli cells *via* GJ/desmosome. More advanced spermatids (steps 8 and later) are connected to Sertoli cells *via* apical ectoplasmic specialization. The BTB, which is created by two adjacent Sertoli cells, divides the seminiferous epithelium into two compartments: adluminal and basal. At stage VIII of the epithelial cycle, the “new” BTB forms before the “old” BTB disassembles to allow the transport of preleptotene/leptotene spermatocytes. Also, near the seminiferous tubule lumen, apical ectoplasmic specialization disassembles to release the elongated spermatids during spermiation. The apical ectoplasmic specialization is hypothesized to be associated with microtubule (MT)-based motors (kinesins and dyneins) that allow “down and up” movement in the apical seminiferous epithelium of maturing spermatids.

Mammalian testes produce sperm over a male’s lifetime. Diploid spermatogonia evolve into mature haploid spermatozoa in a cyclic process called spermatogenesis. Each spermatogenic wave is characterized by three distinct functional phases: 1) spermatogonia (2N, 2C) proliferation by mitosis, 2) a single primary spermatocyte (2N, 4C) to yield two secondary spermatocytes (1N, 2C) by meiosis I, followed by four haploid round spermatids (1N, 1C) by meiosis II, and 3) transformation of spermatids into streamlined, flagellated, and functionally competent spermatozoa by spermiogenesis (Figure 3) (Griswold, 2016; de Kretser et al., 1998). Mitosis and meiosis, the two successive cell divisions in spermatogenesis, are tightly regulated processes, with the latter being a fundamental element of gametogenesis (Griswold, 2016; de Kretser et al., 1998). Kinesins are involved in spindle morphogenesis, chromosomal alignment and segregation during mitosis and meiosis in mammalian testes. For example, KIF11 (Eg5/kinesin-5) is a known mitotic kinesin that regulates microtubule sliding in mammalian somatic cells to facilitate bipolar spindle assembly (Hara-Yokoyama et al., 2019). During mouse spermatogenesis, KIF11 is closely associated with mitosis and meiosis and can serve as a marker of spermatogenic progression, even in mutant mice with arrested spermatogenesis, by giving a timeline of spindle formation (Hara-Yokoyama et al., 2019). When a specific/selective KIF11 inhibitor such as Monastrol, S-trityl-l-cysteine, or Dimethylenastron was injected into mouse testis, dividing spermatocytes exhibited impaired spindle bipolarity following KIF11 suppression. KIF11 inhibition also causes a reduction in spermatid count and defective sperm in mice (She et al., 2020a). By immunofluorescent staining, KIF10 from kinesin-7 family was revealed to be expressed in germ cells in the mouse testis. Following either intraperitoneal or intratesticular injection of a selective KIF10 inhibitor GSK923295, spermatogenesis in the mouse testis was disrupted, possibly due to a mechanism in which KIF10 malfunction causes problems in chromosome alignment and spindle assembly during meiosis (She et al., 2020b). KIF20A, a member of the kinesin-6 family, was also discovered to be required for central spindle organization and cytokinesis in meiosis, as well as acrosome formation during mouse spermatogenesis, according to the same research group (She et al., 2020c). Despite this, more research into the cellular expression and localization of KIFs in the testis is needed, as many of the KIFs that are known to be expressed in the testis have an ambiguous cellular localization, according to the summary of findings in Table 1. As in the case of KIF11, it remains to be verified whether it is restricted to germ cells (esp. spermatogonia and spermatocytes) rather than somatic Sertoli cells of the mouse testis (Hara-Yokoyama et al., 2019; She et al., 2020a), e.g., by immunoblotting and/or mRNA analysis utilizing purified germ or Sertoli cells isolated from rodent testes.

The relevance of kinesins in cell cycle progression is further demonstrated in studies employing knockout mice (Table 2). KIF18A (Kinesin-8 family) activity is required for correct chromosome congression during cell division, and its absence in mice causes significant abnormalities in microtubule dynamics, spindle integrity, and checkpoint activation, resulting in germinal cell aplasia (Liu et al., 2010). Understanding the temporal and spatial expression patterns of KIFs in the testis (e.g., before or after meiosis, as well as expression and intensity in different germ cell types) may also help to elucidate their role in the cell cycle. Mammalian kinesin-1 subtypes [KLC (KLC1, 2 and 3) and KHC (KIF5A, B, and C)], for example, exhibit highly distinct spatiotemporal testicular patterns. KLC3 and KIF5C are expressed in spermatids after meiosis, whereas KLC1, KLC2, KIF5A, and KIF5B are expressed prior to meiosis, with KIF5A identified in somatic testicular cells and the others in spermatocytes (Junco et al., 2001). This suggests that different combinations of kinesin-1 subunits may function at different stages of germ cell differentiation. For a more comprehensive review regarding the role of kinesins in mitosis and meiosis (including non-mammalian), please see (Ma et al., 2017).

### Spermiogenesis

The morphogenesis of the sperm head and flagellum are two important aspects of spermiogenesis, and the shaping of the spermatid head begins with the formation of the acrosome. The acrosome is a Golgi-derived secretory organelle that covers the anterior half of the sperm head. It is a cap-shaped structure that contains digestive enzymes needed for fertilization. The F-actin-based acroplaxome in the subacrosomal region, as well as the transitory microtubule- and F-actin-containing manchette that envelops the spermatid nucleus, are all part of the spermatid head shaping machinery and work with the acrosome as an endogenous regulating force to remodel the spermatid head (Kierszenbaum and Tres, 2004; Dunleavy et al., 2019; Khawar et al., 2019). KIFC1, for example, which associates with the manchette and the nuclear membrane, is known to play a role in vesicular trafficking during acrosome biogenesis and nuclear shaping in the rat, possibly through a nucleocytoplasmic transport mechanism (Yang and Sperry, 2003; Yang et al., 2006). In humans, reduced testicular KIFC1 expression has been associated to globozoospermia, a male infertility syndrome typified by the presence of round-headed spermatozoa lacking the acrosome (Zhi et al., 2016). KIF3A, KIF5C, KIF17B, KIFC5A, Kinesin-related protein (KRP)3A, and 3B (KIF6) in acrosome biogenesis and nuclear shaping; as well as KLC3, KIF3A, and KIF17B in sperm tail formation, have been reviewed recently and are not reiterated here (Ma et al., 2017; Pleuger et al., 2020). Multiple KIFs are believed to be related to the manchette, including KLC3, KIF3A, KIF3B, KIF5C, KIF10, KIF17B, KIF27, and KIFC5A (Ma et al., 2017; She et al., 2020b; Pleuger et al., 2020).

It is conceivable that large amounts of spermatid/sperm proteins must be synthesized due to the massive morphological and structural changes that occur during spermiogenesis. A highly specialized approach to postmeiotic gene expression has evolved in the testis. During mid-spermiogenesis, for example, the nucleus enters a transcriptional pause, and mRNAs required for spermatid differentiation are saved for later use until the nucleus resumes translation in elongate spermatids. Premature mRNA translation, which interrupts spermiogenesis, causes fertility problems (Hecht, 1998; Braun, 1998; O'Donnell, 2014; Geisinger et al., 2021). KIF17B, a testis-specific and enriched kinesin, has been demonstrated to be crucial for CREM (cAMP-responsive element modulator, a transcriptional activator)-dependent transcription and translation in spermatids. In the mouse testis, KIF17B functionally interacts with the transcriptional coactivator ACT (activator of CREM in testis) (Macho et al., 2002; Kotaja et al., 2005; Hogeveen and Sassone-Corsi, 2006) and mRNAs of ACT/CREM target genes (RNA-binding proteins such as TB-RBP and Miwi, which bind directly to KIF17B, form complexes with those mRNAs and act as adaptor, repressor, and/or stabilizer proteins) (Deng and Lin, 2002; Chennathukuzhi et al., 2003; Hogeveen and Sassone-Corsi, 2006; Kotaja et al., 2006), and KIF17B regulates their nucleocytoplasmic trafficking in post-meiotic spermatids. Thus, by attaching or detaching such gene expression elements and transferring them between the nucleus and the cytoplasm, KIF17B modulates the time of transcription termination and/or translation initiation. In the case of the ACT, KIF17B shuttle is driven by protein kinase A (PKA)-dependent phosphorylation rather than a motor- or microtubule-dependent mechanism, as deletion of the motor domain or disruption of the cellular microtubule network have no effect on KIF17B mobility (Kotaja et al., 2005; Hogeveen and Sassone-Corsi, 2006).

## Germ Cell Transport Across the Seminiferous Epithelium

### Sertoli-Germ and Sertoli-Sertoli Cell Junctions in the Seminiferous Tubules

Unlike motile cells, male germ cells do not have the motile appendages formed by dynamic actin structures, such as lamellipodia and filopodia in fibroblasts; therefore, they are non-motile and metabolically inactive before exiting the seminiferous tubule to reach the epididymis. Sertoli cells are the “mother” or “nurse” cells that are entirely responsible for the nourishment and transfer of germ cells. Characterized by a teardrop-shaped nucleus and an irregular cell outline, the Sertoli cell stretches from the basement membrane to the lumen of the seminiferous tubules and contains 30–50 germ cells at various stages of differentiation (Figure 3). In rodent testes, spermatogonia, spermatocytes, and steps 1–7 round spermatids are connected to Sertoli cells via intermediate filament-based desmosomes and/or actin-based gap junctions (Pointis and Segretain, 2005; Kopera et al., 2010; Mruk and Cheng, 2011; Xiao et al., 2014a; Rode et al., 2021). When the nucleus polarizes to one side of the cytoplasm and the acrosomal region makes contact with the cell surface (typically the basal side facing the basement membrane), step 8 round spermatids in stage VIII tubules begin to elongate (O'Donnell, 2014), and the desmosome/gap junction is replaced with a type of actin-based adherens junction (AJ) unique to the testis known as apical ectoplasmic specialization (Grove and Vogl, 1989; Vogl et al., 2000; Lee and Cheng, 2004; Cheng and Mruk, 2010a). The apical ectoplasmic specialization disintegrates at the end of spermiogenesis, allowing mature sperm to be released from the seminiferous epithelium at spermiation (O'Donnell, 2014). Throughout their development, the germ cells differentiate while remaining attached to the Sertoli cell, and substantial crosstalk occurs at the Sertoli-germ cell junctions to aid in germ cell trafficking across the seminiferous epithelium (Mruk and Cheng, 2004; Wu et al., 2020).

The blood-testis barrier (BTB), which is created by the basolateral borders of two neighboring Sertoli cells, is another location with complex cell junction dynamics (Figure 3). BTB is an ultrastructure composed of different types of junctions that coexist and interact with one another, namely tight junctions, basal ectoplasmic specialization [although ultrastructurally identical to the apical ectoplasmic specialization, it exhibits differences; for a review, see (Lee and Cheng, 2004)], desmosomes, and gap junctions (Cheng and Mruk, 2012). The BTB physically splits the seminiferous epithelium into two different compartments: adluminal and basal. A-single (As) spermatogonia present as spermatogonial stem cells (SSCs) in the basal compartment. After several rounds of mitosis, type B spermatogonia are produced, followed by preleptotene/leptotene spermatocytes, the only types of germ cells transported apically through the BTB at stages VIII-IX of the seminiferous epithelial cycle. Therefore, the BTB must undergo cyclic remodeling at the appropriate time to ensure that primary spermatocytes enter the adluminal compartment from the basal compartment without compromising the integrity of the BTB (Figure 3) (Dym and Fawcett, 1970; Clermont, 1972; Russell, 1977; de Rooij, 2001; Cheng and Mruk, 2012).

Cell cycle progression and sperm morphogenesis are proposed to be synchronized with germ cell movement and junction restructuring during the seminiferous epithelial cycle of spermatogenesis (Cheng and Mruk, 2010b; Lie et al., 2009). Very little is known about the role of kinesins in these orchestrated processes. In the aforementioned nucleocytoplasmic transport of ACT, KIF17B migrates into the cytoplasm with ACT at the commencement of spermatid elongation around stages VIII-IX of the seminiferous epithelial cycle in the mouse testis, and its cytoplasmic localization is determined by PKA-dependent phosphorylation (Kotaja et al., 2005). At the same stages as KIF17B relocation, a series of coordinated cytoskeletal and junctional events occur simultaneously in the epithelium: 1) disassembly of the apical ectoplasmic specialization for spermiation; 2) assembly of the new apical ectoplasmic specialization at the step 8 spermatid-Sertoli cell interface; and 3) BTB reorganization with “new” BTB assembly and “old” BTB disassembly for spermatocyte transport across the BTB (Figure 3). The cell junctions and cytoskeletons undergo significant turnover, as does communication between Sertoli and germ cells, necessitating extensive and timely transport of proteins, mRNAs, organelles, and vesicles (Cheng and Mruk, 2010a; Cheng and Mruk, 2012). PKA activation has been shown to perturb the Sertoli cell permeability barrier *in vitro*, suggesting that it may contribute to the breakdown of the “old” BTB (Li et al., 2001). Although KIF17B has proven to be a versatile kinesin, its involvement in these signaling events has yet to be characterized, such as whether its translocation and phosphorylation by PKA trigger any cytoskeletal and junctional events. Another such situation is KIF5B. According to a study (Sehgal et al., 2014), KIF5B couples with KLC1 in the mouse testis. KIF5B deficiency induces male sterility, while its knockdown substantially affects testicular cell adhesion. KIF5B regulates plakoglobin transport to the site of desmosome formation in the mouse testis by forming a complex with 14-3-3γ. The desmosome is a form of intermediate filament-based anchoring junction in the testis that is less well understood. As previously noted, desmosomes are located between Sertoli cells at the BTB, as well as between Sertoli cells and germ cells at early stages, such as spermatogonia and spermatocytes (Mruk and Cheng, 2011). Correspondingly, KIF5B and KLC1 were discovered to be expressed in spermatocytes prior to meiosis in mice and rats (Junco et al., 2001). A recent study, however, demonstrated KIF5B to be present in mouse Sertoli cells, where it co-localized with vimentin intermediate filaments (Jung et al., 2019). Therefore, in addition to identifying whether KIF5B/KLC1 is an integrated component of the BTB and/or confers cell adhesion of the spermatocytes to Sertoli cells, more research is needed to examine whether KIF5B plays any role in synchronization of the spermatocyte differentiation and its transport across the seminiferous epithelium (e.g., regulating the cell cycle of spermatocytes *vs* initiating “new” desmosome formation at the BTB and/or between Sertoli cells and spermatocytes).

### Germ Cell Transport Supported by Actin- and Microtubule-Based Cytoskeletons

The cytoskeleton, which includes actin, microtubule, intermediate filament, and septin networks, is essential for the formation and disassembly of Sertoli-germ and Sertoli-Sertoli cell junctions, as well as the regulation of germ cell development and transport across the seminiferous epithelium (Dunleavy et al., 2019; Wu et al., 2021a). Although the role of intermediate and septin filaments in the testis has not been thoroughly studied, there is a better understanding of the actin- and microtubule-based cytoskeletons, which we will discuss below.

During mammalian spermatogenesis, the timely transport of a wide range of organelles and vesicles, including developing germ cells, across the seminiferous epithelium is vital, and it is largely accomplished by microtubule and actin tracks and their associated motors (kinesin, dynein, and myosin superfamilies). Long-distance longitudinal transport of cellular cargoes is assumed to be propelled along microtubule paths, whereas short-distance transverse transport is facilitated by F-actin. Sertoli cells feature massive networks of microtubule and F-actin at different stages of the epithelial cycle to ensure proper cargo delivery (Dunleavy et al., 2019; Hirokawa et al., 2010; Wu et al., 2021b; Barlan and Gelfand, 2017; Wen et al., 2016). F-actin was used for attachment by cell junction proteins at the apical ectoplasmic specialization, as well as tight junction, basal ectoplasmic specialization, and gap junctions at the BTB. At stage VIII of the seminiferous epithelial cycle, transport of preleptotene/leptotene spermatocyte from the basal to the adluminal compartment is dependent on the restructuring of Sertoli cell junctions at the BTB to guarantee meiosis at stage XIV in the rat or XII in the mouse (Figure 3). F-actin tracks across the seminiferous epithelium are only most visible in late stage VIII tubules, implying that actin-based traffic is likely transient (Wang et al., 2020a; Chenouard et al., 2020). Microtubule bundles, on the other hand, run perpendicular to the basement membrane (parallel to the direction of spermatid transport) and stretch over the entire seminiferous epithelium during various stages of the epithelial cycle, with the plus ends at the base and the minus ends at the apex of the Sertoli cell. Microtubule tracks are used for spermatid transport and are intimately linked to the apical ectoplasmic specialization. Developing spermatids travel “down and up” the seminiferous epithelium in the adluminal compartment before returning to the apical regions for spermiation. This is considered to mirror the movement of apical ectoplasmic specialization along the nearby microtubule by engaging either plus or minus end motor proteins (Figure 3) (Dunleavy et al., 2019; Wen et al., 2016; Tang et al., 2016b; Tang et al., 2015; Vaid et al., 2007; Guttman et al., 2000; Beach and Vogl, 1999). Indeed, several kinesins have been implicated in spermatid translocation in the rat/mouse testis, including KIF6/KRP3, KIF20, and KIF15, since they appear to be associated with apical ectoplasmic specialization, elongating spermatids, and/or Sertoli cells (Table 1) (Zou et al., 2002; Vaid et al., 2007). However, functional interactions between these kinesins and components of the apical ectoplasmic specialization can be tested using assays like co-immunoprecipitation to refine the findings. The microtubule track is critical for the delivery of elongated spermatids to the tubule lumen and for the timely completion of spermiation. It is involved in disruption of the apical ectoplasmic specialization upon sperm release (Li et al., 2017; Dunleavy et al., 2019). Furthermore, a major microtubule minus end-directed motor protein, Dynein 1, has been shown to maintain proper F-actin structure in cultured primary Sertoli cells and in adult rat testes. This demonstrates that microtubules also contribute to the integrity of the F-actin network in the testis (Wen et al., 2018).

Kinesins are emerging as regulators or components of cytoskeletons other than microtubules, indicating the coordination of multiple cytoskeletal systems. According to a study combining an *in vitro* model system and single-molecule techniques, kinesin may collaborate with myosin to deliver a specific cargo on both microtubule and actin cytoskeletal tracks (Ali et al., 2008). In *Xenopus* oocytes, a nuclear and meiotic actin-bundling kinesin (NabKin) directly links microtubules with F-actin and coordinates the interactions of these two cytoskeletons. Its somatic paralogue is the actin-binding domain-containing kinesin KIF14 (Samwer et al., 2013). Septin 9, a microtubule-associated septin, has been uncovered to affect interactions between KIF17 and its membrane cargo, as well as the dendritic entry of KIF5 and KIF1 in neurons (Bai et al., 2016; Karasmanis et al., 2018). It has long been known that conventional kinesin is essential for the interplay of vimentin intermediate filaments with microtubules in various cells (Gyoeva and Gelfand, 1991; Liao and Gundersen, 1998; Prahlad et al., 1998). Research into mammalian testes, on the other hand, is still in the early stages. A recent study has shown that, KIF15 knockdown not only impairs Sertoli cell barrier function *in vitro*, but also disturbs the homeostasis of the four cytoskeletons in Sertoli cells: microtubule, actin, vimentin, and septin (Wu et al., 2021a), demonstrating that kinesins have a more profound and extensive role in mammalian testis with its regulatory effects that extend beyond microtubules. Whether kinesins are required for germ cell transport facilitated by multiple cytoskeletons, as well as how kinesins select which track to use and promote cytoskeleton coordination in the testis, merit further investigation.

## Kinesins in Regulation of Epithelial Junctions

Sertoli cell junctions at the BTB and spermatid adhesion onto Sertoli cells, as stated previously, underlie mammalian spermatogenesis and confer Sertoli cell function in the basal and adluminal compartments, respectively. Although the evidence for KIF participation in BTB and apical ectoplasmic specialization is sparse, other epithelial cells, cancer cells, and different migratory cells can provide insight.

Focal adhesion (FA) or focal contact links the internal actin cytoskeleton of an interacting cell with the extracellular matrix (ECM), which is important to cell adhesion and migration. FAs are large, dynamic protein complexes comprising transmembrane integrins and their ECM ligands (such as fibronectin, collagen and laminin), as well as a number of adaptor, scaffold, docking, and signaling proteins between integrins and F-actin [such as talin, vinculin, paxillin, p130Cas, non-receptor tyrosine kinases FAK (focal adhesion kinase) and SFKs (SRC family kinases)], all of which are involved in the dynamic association with the actin cytoskeleton (Wozniak et al., 2004; Mitra et al., 2005; Martino et al., 2018). KIF5A enhances the transport and secretion of type I collagen for FA assembly, according to recent research in lung adenocarcinoma cells (Tan et al., 2022). To increase endoplasmic reticulum (ER) transport to the cell surface and boost FA growth and maturation during cell migration, KIF5B forms a complex with the ER-resident protein kinectin-1 and the small GTPase Rab18 in human osteosarcoma epithelial cells (Guadagno et al., 2020). KIF13A has an impact on FA dynamics in lung adenocarcinoma cells (Banerjee et al., 2021). Various miRNAs can control KIFs to support FA function in gastric cancer cells (Ma et al., 2021). The testis, on the other hand, lacks focal contact. But the same FA component proteins, such as FAK, SFKs, integrins, and laminins, have been identified at the Sertoli-germ and Sertoli-Sertoli cell junctions in the rat testis. The presence of laminin and collagen chains in the basement membrane of the seminiferous tubule has also been reported in new research (Li et al., 2021). FAK and SRC, for instance, are found in the tight junctions and basal ectoplasmic specialization at the BTB, as well as the apical ectoplasmic specialization at the spermatid-Sertoli cell interface. Both FAK and SRC are structurally coupled with the occludin/ZO-1 adhesion complex located at the BTB. The α6β1-integrin/laminin-α3β3γ3 complex is the major component of the apical ectoplasmic specialization, and FAK physically interacts with β1-integrin, which is important during spermiation for sperm release from the seminiferous epithelium (Cheng and Mruk, 2010a; Cheng and Mruk, 2012). This is analogous to how FAK and SRC collaborate to promote FA disassembly in other epithelia or cancer cells (Mitra et al., 2005).

KIF22 binds to CAR (coxsackievirus and adenovirus receptor) and its regulation of microtubule dynamics influences CAR localization at the lung cancer cell-cell junctions (Pike et al., 2018). KIF26A modulates FA and cell adhesion possibly via its effect on protein levels of FAK/p-FAK, E-cadherin/N-cadherin, and p-β-catenin in gastric cancer and mesenchymal cells. KIF26A has also been demonstrated to interact directly with FAK and to drive a pathway that inhibits integrin-SFK-FAK pathway signaling in primary cultured neurons and mouse brains (Combes et al., 2015; Wang et al., 2018; Ma et al., 2021). KIF17 not only stabilizes microtubule, but also enhances cell-cell adhesions by boosting apical actin network assembly and junctional E-cadherin accumulation, and this activity is independent of microtubule binding in epithelial cells (Acharya et al., 2016; Kreitzer and Myat, 2018). While not being engaged in E-cadherin transport, KIFC3 reduces E-cadherin degradation by recruiting the ubiquitin-specific protease 47 (USP47, which deubiquitinates E-cadherin) to AJ, which prevents E-cadherin degradation and maintains the epithelial integrity (Meng et al., 2008; Sako-Kubota et al., 2014). In cultured epithelial cells, kinesin-1 is abundant in the apical junctional complex (AJC), and it similarly accumulates at the epithelial cell-cell contacts in normal human colonic mucosa, where it is coupled with the E-cadherin-catenin complex (Ivanov et al., 2006). Desmoglein-2 (DSG2) and desmocollin-2 (DSC2) are transported by kinesin-1 and -2, respectively, for desmosome assembly in epithelial cells (Nekrasova et al., 2011). KLC and KIF3A/B have both been implicated in endothelial/epithelial barrier function, with the latter involved in tight-junction protein transport and interaction with β-catenin (Wu et al., 2007; Aguado-Fraile et al., 2012; Giridhar et al., 2016; Kreitzer and Myat, 2018; Ichinose et al., 2019; Johansson et al., 2019; Stevens et al., 2020). These integral membrane proteins (CAR, E-cadherin/N-cadherin, DSG2/DSC2), as well as the signaling and adaptor proteins, are all established integrated components of the BTB and apical ectoplasmic specialization (Wu et al., 1993; Wang and Cheng, 2007; Cheng and Mruk, 2010a; Lie et al., 2010; Cheng and Mruk, 2012). As a result, it is worth investigating whether similar interactions occur in the testis. These findings illustrate that a single KIF is likely to favor a specific mode of cargo delivery and/or junction formation, as well as highlight the regulatory effects of KIF on cytoskeletons based on microtubule, actin, and intermediate filaments.

## Kinesins in Human Male Reproduction

The involvement of kinesin in reproduction is an underappreciated topic that is only getting started. There is even less information available about humans. However, growing evidence indicates that kinesins can have an important role in human male reproduction. By analyzing Gene Expression Omnibus (GEO) datasets, KIF2C, and related miRNAs have been connected to spermatogenetic failure and non-obstructive azoospermia in patients (Cao et al., 2021). KIFC1 is linked to globozoospermia in human testes as previously mentioned (Zhi et al., 2016). KIFC1 and KIF3B are enriched in tissues of human seminoma, one of the most frequent testis cancers affecting young men, with a role in cell division regulation in seminoma (Xiao et al., 2017; Shen et al., 2017). Several kinesins, including KIF4, KIF5A, and KIF21B, have been proposed as candidates for human immunopathogenesis (Bernasconi et al., 2008; Alcina et al., 2010; International Multiple Sclerosis Genetics Consortium (IMSGC), 2010), but their contribution to male autoimmune infertility has not been established. On the other hand, KIF1C has been reported to confer dominant resistance to experimental autoimmune orchitis (EAO) in mice, which remains to be studied in human males (del Rio et al., 2012). KIF1A is predominantly expressed in the brain and nerves, and knockout mice died within 1 day of birth (Table 2). KIF1A-related disorders are relatively rare in the general population, but males with pathogenic KIF1A variations have been observed to have tiny testes or penises, as well as cryptorchidism (Kaur et al., 2020; Boyle et al., 2021; Pennings et al., 2020). Similarly, knockout mice for KIF7 died at birth (Table 2), and a boy with acrocallosal syndrome (ACLS) who carries a novel homozygous KIF7 nonsense mutation had unilateral maldescensus testis (Ibisler et al., 2015). These findings suggest that kinesins play a crucial role in human testis development, while it may be difficult to discern their functions from the phenotypes of experimental animal models. Cell lines or cultured primary cells obtained from humans could be a viable alternative to animal models, providing mechanistic insights without demanding fresh primary cultures from human testes. TCam-2, a human tumor cell line derived from a primary testicular seminoma of a 35-year-old men and assumed to reflect human male germ cells, was utilized to investigate KIF18A activity in proliferation, cell cycle and apoptosis in the human cell line (Smialek et al., 2020). The use of a well-established human Sertoli cell *in vitro* system sourced from cadaveric testes of males aged 12–36 years could be a useful tool for studying the role of kinesins in human Sertoli cell function and BTB dynamics (Chui et al., 2011; Xiao et al., 2014b; Chen et al., 2017).

## Concluding Remarks

Herein, we review current findings on kinesins in mammalian spermatogenesis, including their role in cell cycle and sperm morphogenesis, as well as their putative function in germ cell transport through the BTB and the remodeling of cell junctions and underlying cytoskeletons to expedite germ cell transport across the seminiferous epithelium. We also propose a role of kinesins as mediators and/or synchronizers of cell cycle progression, germ cell transport, and junctional turnover in mammalian testes. Yet many aspects of kinesin function in the mammalian testis remain unexplored, such as the mechanism(s) by which they affect spermatogenesis globally and promote Sertoli-germ cell interactions during spermatogenesis. The loss of function of many kinesins resulted in lethality as well as infertility or subfertility in mice (Table 2), but the underlying pathway(s) or molecule(s) is unknown. Nonetheless, it might be able to help us better grasp the role of kinesins in human disorders or as disease markers, providing insight into male factor infertility and male autoimmune diseases. *In vitro* culture of germ cells (or germ cell-like cells) and Sertoli cells derived from human males is also a good supplement to animal model studies.
